# Why ethical frameworks fail to deliver in a pandemic: Are proposed alternatives an improvement?

**DOI:** 10.1111/bioe.13202

**Published:** 2023-07-13

**Authors:** Chris Degeling, Jane Williams, Gwendolyn L. Gilbert, Jane Johnson

**Affiliations:** ^1^ Australian Centre for Health Engagement, Evidence and Values, Faculty for the Arts, Social Sciences and Humanities University of Wollongong Wollongong New South Wales Australia; ^2^ Sydney Institute for Infectious Diseases University of Sydney Sydney New South Wales Australia; ^3^ Department of Philosophy Macquarie University Sydney New South Wales Australia

## Abstract

In the past decade, numerous ethical frameworks have been developed to support public health decision‐making in challenging areas. Before the COVID‐19 pandemic began, members of the authorship team were involved in research programmes, in which the development of ethical frameworks was planned, to guide (a) the use of new technologies for emerging infectious disease surveillance; and (b) the allocation of scarce supplies of pandemic influenza vaccine. However, as the pandemic evolved, significant practical challenges emerged that led to our questioning the value of these frameworks. We now believe that a normative instrument, such as a framework, cannot adequately or reliably provide the ethical guidance that needs to be incorporated into public health decision‐making during natural disasters or infectious disease emergencies. Recently it has been suggested that there are potentially more dynamic, flexible, and effective ways to navigate decisions involving complex considerations entailed in policies and practices during a public health emergency. In this paper, we first outline the key functions of a public health ethics framework, before describing why we believe it would not be fit for purpose during a crisis. We end by considering whether proposed alternative methods to promote ethical public health decision‐making goals have the potential to meet these objectives.

## 1 THE ROLE OF ETHICAL FRAMEWORKS IN PUBLIC HEALTH DECISION‐MAKING

Public health ethics (PHE) is the basis for the analysis of moral problems in public health settings. It incorporates evidence, values, ethical argument, and moral principles to assess the justifiability of public health actions or to help shape optimal public health practice. Like all other forms of public policy, decision‐making in public health is an exercise in resource allocation, which is then implemented and governed by relevant organisations through laws, regulations, and social structures.^1^ Public health also has a role in determining which social norms to promote or reinforce, and PHE aims to bring ethical considerations into policy deliberations around prompts for action. Accordingly, the discipline of PHE focuses on two key dimensions of how norms are prioritised and resources allocated:

(i) whether the goals and priorities of public health policies and programmes promote fairness or a particular conceptualisation of justice (commonly construed in policy circles as a trade‐off between the efficiency and equity of interventions); and
(ii) whether the goals and practices of public health policies and programmes, can be considered legitimate and justifiable expressions of state power (commonly construed in ethical debates as a trade‐off between sustaining public goods and protecting individual liberty).

Within these boundaries, an explicit goal of PHE is to bridge the gap between ethical theory and public health practice in circumstances where the state potentially has an interventionist role. PHE is different from medical ethics and bioethics because it focuses on the role of governments in promoting ‘health’ and takes the *population* as its unit of concern. Typically, the ethical challenges confronted by PHE are distinct from those facing individual practitioners and health service users in clinical settings. However, during a global public health crisis the concerns of medical ethics and PHE inform each other and can become more closely entangled, as the COVID‐19 pandemic has shown.

Against this background, decision‐making frameworks are central to the disciplinary identity of PHE and have, to an extent, shaped its parameters.^2^ Ethical frameworks can be described as pragmatic devices designed to explicate the values, principles or issues relevant to public health decisions.^3^ They rest on assumptions about the value of scientific evidence for informing rational processes of policy‐making. Such frameworks are not well‐defined; they can be interrogative, algorithmic, or a mixture of both. Early influential frameworks tended to be interrogative and pose questions about how to incorporate certain principles (e.g., the principle of the least restrictive means), considerations (e.g., distributing burdens and benefits fairly) or values (e.g., reciprocity and solidarity) for consideration by decision‐makers. Typically, these types of ‘laundry list’ frameworks can lead to a range of ethically justified conclusions. More recently, algorithmic or protocol‐based frameworks have been developed to provide decision‐makers with a final ‘answer’ for specific ethical problems such as the rationing of scarce resources.^4^

When operating as intended, ethical frameworks allow decision‐making to become detached from debates about normative theory, and thereby to side‐step discussions around whether and how different measures or proposals can be ethically justified.^5^ Underlying early framework development was the belief that the key issue in PHE was brokering a compromise between individual rights and the public good.^6^ Following encouragement from the WHO, in the last decade, governments around the world sought to develop ethical frameworks to guide decision‐making in communicable disease control and prevention.^7^

The rapid emergence and global spread of SARS‐CoV‐2 has amply demonstrated that pandemics are public health emergencies that require ethically informed decision‐making in circumstances of acute and dynamic uncertainty.^8^ The burden of infection, morbidity and mortality in communities can escalate quickly, unequally and unpredictably. Consequently, there may not be adequate or reliable evidence to inform key decisions about whether to implement, and/or how to target, measures that restrict established civil liberties, or limit access to scarce resources. Policy formulation and implementation always take place in a political context where the evaluation of evidence of ‘what works’ is legitimately assessed against the need to protect the interests of a diverse range of stakeholders.^9^ In these circumstances the unifying goal is not, necessarily, to acquire high‐level evidence about practical barriers and potential effects, but to develop context‐specific ‘evidence enough’ to both justify and guide action in the face of uncertainty.^10^ Given these difficult conditions for decision‐making, an ethical framework should, ideally, be an essential tool for the development of an appropriate and proportionate public health response to a specific problem and context or situation. As Lewis and Schuklenk have argued, ethical frameworks, to be effective, must not only transparently justify consistent decision‐making, but also guide adequate public health action.^11^

During the current pandemic, several frameworks have been proposed, including for vaccine distribution,^12^ clinical resource allocation,^13^ the use of contact tracing and screening technologies^14^ and the rationing of personal protective equipment.^15^ Arguably, many have failed to live up to their promise.

Our research team includes members with expertise in public health and empirical bioethics, health social science, field philosophy and infectious disease practice and policy. Since 2015, we have been involved in government‐funded projects to develop ethical frameworks to guide decision‐making for pandemic responses and technologically enhanced communicable disease surveillance. In 2020 and 2021, as new dilemmas and challenges involved in COVID‐19 pandemic management became central to government policy formation, the value of such frameworks seemed increasingly questionable. In subsequent discussions with peers, and examination of evolving commentary on the ethics of pandemic management,^16^ it became apparent that our impressions and experiences were shared by others. Recently, teams led by Greenhalgh^17^ and Lancaster^18^ have argued for a more pragmatist approach to public health decision‐making, including the processes whereby ethical considerations inform policy deliberations. In this paper, we elaborate on our reasons for questioning the value of ethical frameworks and consider other ways in which ethical considerations can be embedded into context‐appropriate public health decision‐making when evidence is dynamic and uncertain.

## 2 CHALLENGES FOR IMPLEMENTING AN ETHICAL FRAMEWORK IN A PANDEMIC

### 2.1 Pitching the framework at the right level

It is difficult to design an ethical framework, in advance, that is pitched at the right level when decision‐making and implementation are required, in practice. Many existing influential frameworks are intended to be applicable to a wide variety of public health situations. The concept of a single framework that can be applied to a range of interrelated public health problems is intuitively appealing. Yet, as the complexity of policy‐making in the current pandemic has shown, designing an effective ethical framework requires in‐depth knowledge or substantive experience of the practical context in which decision‐making will be applied, and how decisions, in one context or domain of practice, can have flow‐on effects in others.

For example, during the first wave of the SARS‐COV‐2 pandemic in Northern Italy, ethical frameworks and prioritisation criteria for the allocation of limited clinical resources were imposed on healthcare professionals.^19^ In contrast, some hospitals and healthcare providers did not assume conditions of scarcity and competition, but improvised ways to share intensive care beds and provide effective care, collaboratively, thereby avoiding many life‐and‐death decisions.^20^ According to Pascoe and Stripling, the development and dissemination of scarce resource allocation frameworks contributed to a *disaster imaginary* that unhelpfully cast older people as dispensable in the pursuit of the greater good.^21^ In complex and evolving situations, appropriate ethical guidance often depends on an appreciation of the specific (as well as the broader) context, granular knowledge of technical detail, or both. Without such clarity, knowledge and experience, anticipatory ethics can easily fail. A framework can be too general—that is, it lumps together disparate things that need to be kept separate—leading to unintended and/or perverse outcomes. Or, equally, a decision‐making framework can be so narrowly focussed and specific that it cannot be extrapolated to other settings and/or it inhibits contemplation of unanticipated alternative strategies. When evaluating the fairness and legitimacy of different courses of action, specific public health decisions and the social, cultural, and political contexts of proposed interventions matter, and cannot always be accurately predicted.

To be useful to decision‐makers an ethical framework should provide clear, practical guidance so that it stands alone as a functional guide that provides transparent, consistent justifications for actions. To fulfil this role, decision‐makers must know it exists and be willing to be educated about how to use it appropriately. If they are unfamiliar with how to integrate the framework into their deliberations, its purpose may not be apparent. For example, it is often unclear whether a framework is intended to ensure acceptable decision‐making processes or optimal outcomes.^22^ Therefore, clarity about the aims, intended range of applications, and procedures for the implementation of a framework is essential to its coherence, consistency, relevance, and usefulness.

How people, who make or are affected by decisions, respond to the framework is also a critical factor. Ethical arguments for one intervention over another will only receive as much support as the often controversial and unarticulated moral theories on which the framework is based.^23^ By prompting prioritisation of some values, frameworks articulate what matters most, whether or not this is explicitly stated.^24^ Prior communication of the embedded or expressive values of a framework is essential to ensure that those who use or are impacted by, decisions shaped by it, understand the implications. Ethical guidance must be justified and published widely; it should not be a ‘policy makers’ “dirty” secret’.^25^ Without prior broad and inclusive consultation with all relevant stakeholders, it may be politically difficult for decision‐makers to use the framework. On the other hand, it may be impossible to pitch a framework in advance and also take account of the rapidly changing needs of decision‐makers.

### 2.2 Accommodating rapidly changing facts, circumstances and priorities

Even in the context of an entirely new emerging infectious disease (EID) outbreak or pandemic, there are usually some predictable patterns. For example, the precautions required to limit disease transmission can be based on experience from past outbreaks. Failures of effective outbreak control are often due to non‐compliance with established public health or infection prevention and control principles.^26^ Nevertheless, during EID crises, new evidence can emerge quickly—for example, changes in reproductive numbers (*R*_0_/*R*_eff_), morbidity, case fatality rate, transmission routes, disruption to essential services, and so forth—due to pathogen evolution (i.e., SARS‐CoV‐2 variants); new interventions (vaccines and therapies); and the effects of restrictions, such as lockdowns.

Changing circumstances (risks) mean that decisions or actions based on previous risk assessments may require amendments that bring previously aligned beliefs into conflict. For example, long‐established infection prevention and control *principles* will still apply, but different, potentially controversial, *practices* may be needed. Controversy can arise because of differences in the interpretation of evidence or the level of risk tolerance or aversion. During EID emergencies, including the COVID‐19 pandemic, many governments have opted (or been urged) to take a ‘precautionary’ approach such that *saving as many lives as possible*, for example, overrides all other values.^27^ In ideal circumstances, public health decision‐making should be nuanced and grounded in robust evidence. However, when different values and policy goals need to be balanced, economic, scientific and epidemiological evidence may not be sufficient.^28^ Dynamic evidence and uncertainty mean that ethical reasoning must be based on decision‐makers’ incomplete knowledge. Assumptions about risks, or how people will respond to interventions, can be wrong in ways that affect outcomes and so become ethically significant.^29^

When uncertainty arises from an absence of data, ethical analysis may assist, by suggesting a default position that should be taken presumptively, unless or until more evidence becomes available. In the face of a pandemic, decision‐makers and ethicists are forced, to some degree, to trade reliability for speed, such that applying the precautionary principle is intuitively appealing. However, a precautionary approach can cause confusion among decision‐makers because it is usually unclear whether it should be applied as an epistemic or decision rule.^30^ When applied as an epistemic rule, precaution demands that decision‐makers act only when the supporting evidence is clear; whereas, when operating as a decision rule, it demands that actions should be based on the balance of whatever evidence is available, about how to prevent the worst outcome.^31^ Moreover, once applied, a precautionary approach is difficult to roll back and may inhibit more effective responses to emerging circumstances. It may create new risks—such as disruptions to non‐COVID healthcare, education or business; family violence; or loneliness—that are due to the public health responses rather than the emergency per se.^32^

To remain useful as evidence emerges and changes, ethical advice should be able to identify what is relevant *now*, and why some values are more important than others, based on the degree of confidence in the current evidence. In reality, the need to operationalise different priorities and values can be central to considerations in the same public health scenario as it unfolds.^33^ It may not be possible for a single framework to adequately capture and account for all dimensions of the problem. A set of generalised values or questions (as found in many frameworks) does not necessarily address the most pertinent questions relevant to a particular situation but may ask decision‐makers to consider values that are (or will soon become) inconsistent or redundant.

Adding further complications to this temporal feature, the relationship between values listed for consideration and questions posed, or how to apply them to the problem at hand, may be unclear or inconsistent.^34^ For example, it may be difficult to reconcile aims that involve maximising utility with those that focus on equity or justice, when the situation is acute and changing rapidly and the consequences of all choices are tragic. In such circumstances, ethical frameworks and bioethicists’ normative criticisms can cause distress and moral injury to people facing controversial choices.^35^ A framework recommending attention to both maximal utility and equity should flag this potential difficulty and offer ways to reconcile the aims. It is not productive simply to substitute evidence uncertainty with the normative disagreement of a stylised ‘trolley problem’ in the midst of a crisis.^36^ Without a clear process that produces consistent guidance and justifications for any proposed action, ethical frameworks become an impoverished version of ‘principilism’.^37^ These constraints and potential design failures mean that, in dynamic situations, frameworks risk both theoretical and practical incoherence, which can lead to unnecessary burdens for decision‐makers and missed opportunities for meaningful incorporation of ethics into public health planning.

### 2.3 Being clear about who the framework is for

As noted earlier, frameworks frequently offer a set of values or questions that are intended to be used to help assess public health initiatives. This approach is potentially useful in encouraging a more complete or nuanced view of the situation at hand. It is, however, unlikely to be helpful to the range of potential users representing organisations that formulate their own policies during a public health emergency. These might include different levels of government and various departments, hospitals, medical specialities, unions representing essential workers and consumer organisations. Adding to this complexity, some of these actors will have different goals and agendas, and so disagree publicly or operationally with ‘official’ policies, whether or not the latter have been developed with reference to a framework. For example, the Canadian National Advisory Committee on Immunisation (NACI) guidance document, for COVID‐19 vaccine distribution,^38^ did not describe the metaethical standards or principles that guided its formulation.^39^ Priority group recommendations for COVID‐19 vaccination, in the NACI framework, were developed through an established Ethics, Equity, Feasibility and Acceptability (EEFA) process,^40^ after consultation with expert stakeholders and Canadian population surveys.^41^ However, once COVID‐19 vaccines became available and rolled out, in December 2020, the uptake of the NACI prioritisation recommendations varied. In some Canadian provinces frontline workers such as paramedics, firefighters, teachers and transport workers questioned their absence from the prioritisation plan.^42^ Ultimately, as in many high‐income countries, the vaccine rollout in Canada was largely successful but failed to achieve the equity goals at the core of the EEFA framework.^43^

Against this background, ethical frameworks embody a form of institutional rationality founded on assumptions about linearity in policy‐making and the primacy of scientific evidence in effective sociotechnical problem‐solving. Yet, in practice, decision‐makers draw on their expertise, values, biases, and various information sources (scientific studies, media, ideology, experience), to construct a proportionate response. As decision‐making becomes distributed across different stakeholders, a generalised set of values or questions may not address the most pertinent questions for a particular role, or ask decision‐makers to consider values that are not relevant to their responsibilities.

In the absence of context‐specific guidance, some culturally familiar values (e.g., autonomy, broadly construed) are likely to be prioritised over others. For example, in the U.K. government's pandemic‐focussed *Ethical Framework for Adult Social Care* 44, there was an implicit emphasis placed on autonomy. This was problematic because, early in the pandemic, members of the public could not make fully informed decisions about their health risks from COVID‐19^45^; this was especially so for those with reduced cognitive capacity, many of whom were at higher risk of infection and poor outcomes. Differences or incommensurability between the expressive values of a framework and core values of those impacted by associated decisions or implementation measures can have serious implications.^46^ For example, decisions about allocation of ventilators, based on a strong correlation between life expectancy and creatinine scores, were intended to promote the value of preserving the most life‐years, but disadvantaged patients from groups with lower socioeconomic status.^47^ Such discrepancies can lead to the perpetuation of the status quo or to neglect of important but politically difficult issues, such as the role of structural racism and social disadvantage in perpetuating health inequity.^48^

## 3 ALTERNATIVES TO FRAMEWORKS TO GUIDE PANDEMIC DECISION MAKING

Using ethical frameworks to guide actions, under conditions of acute and dynamic pandemic uncertainty, is not only difficult but can be distracting. Frameworks that are inappropriate in a specific decision‐making context can create significant burdens for decision‐makers.^49^ Frameworks can also promote policies that cause unnecessary harm to those affected by decisions, by creating or reinforcing inequities in access to services or adversely affecting health outcomes. Unless the expressive values embedded in the framework are understood, and acceptable to those affected by the resulting decisions, their use can undermine trust in the public health response.

The key ethical issues that need to be considered by decision‐makers, during public health emergencies (e.g., privacy, autonomy, social justice, inclusiveness, trust, transparency) are well‐known. Arguably, a past focus on developing ethical instruments, rather than reflexive practice and normative evaluation of emerging and contingent policy goals, left decision‐makers poorly prepared for the current pandemic. It is now proposed that, instead of inserting a framework that bureaucratises the ethical dimensions of policy‐making processes, similar goals can be achieved by a pragmatist and abductive approach.^50^ In practice, this means that normative analysis informs decision‐making, through consideration of how conflicting values are best addressed. In this context, reasoning is about evaluating alternative perspectives, on the premise that values and ethical concerns only become salient when they guide, and operate in close relation to, current practices. Beyond the shared commitment to promote ‘good’ outcomes, ethical theories do not predetermine practices but become significant to policy deliberations through iteratively engaging with the contextual characteristics of an evolving real‐world situation.

For founding American pragmatists, John Dewey^51^ and Charles Sanders Peirce,^52^ the role of values is to direct action.^53^ Reasoning is not focussed on developing a set of justifications against a predetermined moral system or fixed ends that deaden thinking about emerging events.^54^ In the simplest terms, for the pragmatist, the validity of a value or belief for determining action can be seen in terms of its practical consequences—if we act upon it and our actions turn out well, then that value or belief can be taken as ‘true’ in a pragmatist sense.^55^ Rather than rely on decision‐makers getting the big decisions ‘right’ in difficult circumstances, it has been suggested that pragmatist approaches should promote humility and offer greater reflexivity and flexibility in crisis decision‐making.^56^ As noted by Greenhalgh and Engebretsen, under these conditions both the expected outcomes and unforeseen consequences of decisions need to be identified and considered in subsequent iterations of the policy or response such that ‘knowledge (which is seen as social and contextual) is tested by its ability to help us solve specific problems as they emerge’.^57^ To ensure that decision‐makers consider the ethical dimensions of the situation faced, they need to actively search for creative solutions to the value conflicts entailed by the contextual detail of the specific problem faced.^58^

The extent to which pragmatist approaches should be distributed across decision‐making roles, in organisations involved in a large‐scale public health response, has yet to be specified by those proposing such a change.^59^ Presumably decision‐making/leadership roles could also be decided pragmatically, depending on who is available and appropriately qualified (in context). In any event, they need to be transparent to ensure accountability and avoid confusion about who is in charge. Irrespective of whether the authority to act pragmatically is shared with lower‐level actors such as front‐line public health practitioners and local managers, seeking and responding to feedback is crucial to the success of this type of policy‐making.^60^ Decision‐makers grounded in policy implementation have a greater capacity to explain and respond iteratively to the impacts of an intervention. However, if pragmatist decision‐making is restricted to high‐level officials, they need to communicate the logic and monitor the consequences of their decisions, in consultation with those responsible for their implementation, or affected by them.

A key feature of such an approach is the involvement of experienced advisors to inform and participate in critical decision‐making processes—especially when the stakes are high. Epistemic justice requires that major policy deliberations include, inter alia, representatives of minority and vulnerable groups, and people trained in PHE and bioethics, who can bring values, a historical perspective, and a future vision to the table and who understand the strengths and constraints of the targeted ‘publics’. The need for a broad range of perspectives and methodological pluralism is particularly acute if—as in a pandemic—the real‐world situation is complex, rapidly changing and risky. Even when a policy has been guided by an advisory group, the final decision and responsibility must rest with a responsible public health official. Transparency of process, rationale and responsibility for policy‐making is crucial to public trust and confidence. The experience of the *Ethical Advisory Board* for the U.K.'s NHSX COVID contact‐tracing app indicates that the ethical issues involved in policy decisions, and the policy advisory group's membership, terms of reference and ethical advice must be placed in the public domain; policy implementation must then adhere to the ethical principles specified by the advisory group and agreed to by the responsible government official.^61^

## 4 CONCLUDING CONSIDERATIONS

Arguments for adopting pragmatist approaches to crisis management,^62^ and, more broadly, to enhance democratic policy‐making, are not new.^63^ Evaluations of how pragmatist approaches to crisis decision‐making could work, indicate that, in practice, they could have significant limitations. While ethical frameworks are intended to be useful aids that can be inserted into existing decision‐making processes, the prerequisites for the success of a pragmatist approach are relatively demanding in that bureaucracies, organisations and public need to be both agile and highly responsive to government advice. There also needs to be an administrative capacity to coordinate activities and collect, communicate and analyse feedback so that knowledge and interventions can be adjusted and refined rapidly and iteratively. However, the inherent inertia of organisational systems prevents rapid change. Given this starting point, a pragmatist approach could be inefficient, overly consultative, and time‐consuming. This could make it difficult, politically, for decision‐makers to sustain public trust, during a crisis, unless it is applied selectively only to the most crucial or contentious policies and the process is fully transparent.

Pragmatism is not, necessarily, an antidote to ideology, bias or exclusion of minority perspectives in decision‐making, or to ignorance or non‐compliance during policy implementation. In practice, the fine adjustments required to develop ‘good enough’ knowledge may be difficult to explain, let alone communicate, and inconsistencies in guidance and a sense of crisis may undermine confidence in any proposed action. Crucially, the knowledge accumulation and associated ethical deliberations, embedded at the core of a pragmatist approach, permit what, in retrospect, may look like decision‐making mistakes.^64^ This is less likely to be problematic if the decision‐making process is transparent and the likelihood that policy modifications will be needed is foreshadowed at the start. A recent analysis of government responses to successive COVID‐19 waves, by Boin and Lodge, indicates that administrative and political systems and the broader public are not organised to allow for these types of errors and adjustments in crisis management.^65^

It has been noted that our shared experience of COVID‐19 presented an opportunity to reconstruct the relationship between public and public health—reorganising systems so that the public brings valuable insights and interpretations into policy deliberations.^66^ This requires establishing channels for dialogue between decision‐makers, frontline service providers, vulnerable and minority groups, and the wider community. These channels could take the form of standing committees or advisory groups, such as exist in many countries. Once established, with appropriate representation, a statutory advisory body that routinely provides guidance on policy‐related ethical and cultural issues, could be rapidly engaged during a crisis, with a specific, technical role. A pragmatist approach would be easier to sustain because there would have been time to develop reflexivity, flexibility and credibility. Under this participatory model of policy‐making, decision‐makers and practitioners can make judgments about the ethical appropriateness of actions, in relation to a range of technical, political, and ethical considerations, through a communicative process of dialogue, argumentation and iterative learning. When social scientists and ethicists have been drafted into national‐advisory bodies during the current pandemic, they have often been deemed to have less legitimacy and relevance than members with biomedical expertise.^67^ This is less likely if they have been already accepted as essential contributors to routine policy decision‐making. Compounding the ethical and socio‐cultural knowledge gaps during the pandemic, broader engagement with public and specific groups has typically been *ad hoc* because of emerging crises or policy implementation failure rather than as a necessary preparatory step during policy development.^68^

Reflecting on the lack of integration of ethics into pandemic decision‐making, Emanuel and colleagues note the following: ‘positioning ethics to meaningfully inform decisions requires changing the policy‐making process’.^69^ Clearly, a pragmatist approach is neither a panacea for ethical decision‐making under conditions of complexity and uncertainty nor a ready‐made solution for the limitations of ethical frameworks. Perhaps a key lesson to be drawn from the limitations of both approaches is that there needs to be a flatter hierarchy of expertise during public health crises. For pragmatists like Dewey, the way to introduce appropriate values into decision‐making is to establish transparent systems for the dissemination and communication of the results of social inquiry.^70^ Therefore, processes within which the inclusion of a broad range of perspectives brings all relevant evidence, values and contextual detail into decision‐making should promote better and more values‐informed decision‐making. On this basis, the larger project of ensuring that values and ethical considerations are brought into decision‐making in a public health emergency (and the day‐to‐day governance of public health activities) requires the improvement of the methods and conditions of debate, discussion and persuasion in public and policy deliberations.

## Footnotes

1 Hill, M., & Hupe, P. (2008). *Implementing public policy: An introduction to the study of operational governance*. Sage; Paul, K., & Haddad, C. (2022). The pandemic as we know it: A policy studies perspective on ignorance and nonknowledge in COVID‐19 governance. In M. Gross & L. McGoey (Eds.), *Routledge international handbook of ignorance studies* (2nd ed., pp. 221–233). Routledge.

2 Grill, K., & Dawson, A. (2017). Ethical frameworks in public health decision‐making: Defending a value‐based and pluralist approach. *Health Care Analysis*, *25*, 291–307.

3 Dawson, A. (2009). Theory and practice in public health ethics: A complex relationship. In *Public health ethics and practice* (pp. 191–201). Policy Press.

4 For example, see Savulescu, J., Vergano, M., Craxì, L., & Wilkinson, D. (2020). An ethical algorithm for rationing life‐sustaining treatment during the COVID‐19 pandemic. *British Journal of Anaesthesia, 125*(3), 253–258.

5 Dawson, op. cit. note 3.

6 Kass, N. (2001). An ethics framework for public health. *American Journal of Public Health, 91*, 1776; Childress, J. F., Faden, R. R., Gaare, R. D., Gostin, L. O., Kahn, J., & Bonnie, R. J. (2002). Public health ethics: Mapping the terrain. *Journal of Law, Medicine & Ethics*, *30*, 170–178. https://doi.org/10.1111/j.1748-720X.2002.tb00384.x; Upshur, R. (2002). Principles for the justification of public health intervention. *Canadian Journal of Public Health, 93*, 101.

7 Fairchild, A. L., Haghdoost, A. A., Bayer, R., Selgelid, M. J., Dawson, A., Saxena, A., & Reis, A. (2017). Ethics of public health surveillance: New guidelines. *The Lancet Public Health, 2*(8), e348–e349; World Health Organisation. (2009). *Pandemic influenza preparedness and response: A WHO guidance document*.

8 Boin, A., & Lodge, M. (2021). Responding to the COVID‐19 crisis: A principled or pragmatist approach? *Journal of European Public Policy, 28*(8), 1131–1152; Savulescu, J., Persson, I., & Wilkinson, D. (2020). Utilitarianism and the pandemic. *Bioethics, 34*(6), 620–632.

9 Hill & Hupe, op. cit. note 1.

10 Lancaster, K., & Rhodes, T. (2022). The thing we call evidence: Towards a situated ontology of evidence in policy. In S. Ehlers & S. Esselborn (Eds.), *Evidence in action between science and society: Constructing, validating and contesting knowledge* (pp. 145–164). Routledge.

11 Lewis, J., & Schuklenk, U. (2022). Principlist pandemics: On fraud ethical guidelines and the importance of transparency. In M. Boylan (Ed.), *Ethical public health policy within pandemics* (pp. 131–148). Springer.

12 See: Jecker, N. S., Wightman, A. G., & Diekema, D. S. (2021). Vaccine ethics: An ethical framework for global distribution of COVID‐19 vaccines. *Journal of Medical Ethics, 47*(5), 308–317; WHO. (2020). *WHO/SAGE values framework for the allocation and prioritisation of COVID‐19 vaccination*. https://apps.who.int/iris/bitstream/handle/10665/334299/WHO-2019-nCoV-SAGE_Framework-Allocation_and_prioritization-2020.1-eng.pdf?sequence=1&isAllowed=y; Emanuel, E. J., Persad, G., Kern, A., Buchanan, A., Fabre, C., Halliday, D., Heath, J., Herzog, L., Leland, R. J., Lemango, E. T., Luna, F., McCoy, M. S., Norheim, O. F., Ottersen, T., Schaefer, G. O., Tan, K.‐C., Wellman, C. H., Wolff, J., & Richardson, H. S. (2020). An ethical framework for global vaccine allocation. *Science, 369*(6509), 1309–1312; Liu, Y., Salwi, S., & Drolet, B. C. (2020). Multivalue ethical framework for fair global allocation of a COVID‐19 vaccine. *Journal of Medical Ethics, 46*(8), 499–501.

13 See: Dawson, A., Isaacs, D., Jansen, M., Jordens, C., Kerridge, I., Kihlbom, U., Kilham, H., Preisz, A., Sheahan, L., & Skowronski, G. (2020). An ethics framework for making resource allocation decisions within clinical care: Responding to COVID‐19. *Journal of Bioethical Inquiry, 17*(4), 749–755; Aquino, Y. S. J., Rogers, W. A., Scully, J. L., Magrabi, F., & Carter, S. M. (2022). Ethical guidance for hard decisions: A critical review of early international COVID‐19 ICU triage guidelines. *Health Care Analysis, 30*(2), 163–195; DeJong, C., Chen, A. H., & Lo, B. (2020). An ethical framework for allocating scarce inpatient medications for COVID‐19 in the US. *JAMA, 323*(23), 2367–2368; Lim, S., DeBruin, D. A., Leider, J. P., Sederstrom, N., Lynfield, R., Baker, J. V., Kline, S., Kesler, S., Rizza, S., Wu, J., Sharp, R. R., & Wolf, S. M. (2020). Developing an ethics framework for allocating remdesivir in the COVID‐19 pandemic. *Mayo Clinic Proceedings, 95*(9), 1946–1954; White, D. B., & Lo, B. (2020). A framework for rationing ventilators and critical care beds during the COVID‐19 pandemic. *JAMA, 323*(18), 1773–1774.

14 See: Lo, B., & Sim, I. (2021). Ethical framework for assessing manual and digital contact tracing for COVID‐19. *Annals of Internal Medicine, 174*(3), 395–400; Mbunge, E., Fashoto, S. G., Akinnuwesi, B., Metfula, A., Simelane, S., & Ndumiso, N. (2021). Ethics for integrating emerging technologies to contain COVID‐19 in Zimbabwe. *Human Behaviour and Emerging Technologies, 3*(5), 876–890.

15 See: Binkley, C. E., & Kemp, D. S. (2020). Ethical rationing of personal protective equipment to minimise moral residue during the COVID‐19 pandemic. *Journal of the American College of Surgeons, 230*(6), 1111–1113; Merchant, T., Hormozian, S., Smith, R. S., Pendergrast, T., Siddiqui, A., Wen, Z., & Sheldon, M. (2022). Ethical principles in personal protective equipment inventory management decisions and partnerships across state lines during the COVID‐19 pandemic. *Public Health Reports, 137*(2), 208–212.

16 Christensen, T., & Lægreid, P. (2020). Balancing governance capacity and legitimacy: How the Norwegian government handled the COVID‐19 crisis as a high performer. *Public Administration Review, 80*(5), 774–779; Saxena, A., Bouvier, P. A., Shamsi‐Gooshki, E., Köhler, J., & Schwartz, L. J. (2021). WHO guidance on ethics in outbreaks and the COVID‐19 pandemic: A critical appraisal. *Journal of Medical Ethics, 47*(6), 367–373; Pascoe, J., & Stripling, M. (2020). Surging solidarity: Reorienting ethics for pandemics. *Kennedy Institute of Ethics Journal, 30*(3), 419–444; Emanuel, E. J., Upshur, R. E., & Smith, M. J. (2022). What Covid has taught the world about ethics. *New England Journal of Medicine, 387*(17), 1542–1545; Lewis & Schuklenk, op. cit. note 11.

17 Rutter, H., Wolpert, M., & Greenhalgh, T. (2020). Managing uncertainty in the Covid‐19 era. *BMJ, 370*, m3349; Greenhalgh, T., & Engebretsen, E. (2022). The science‐policy relationship in times of crisis: An urgent call for a pragmatist turn. *Social Science & Medicine, 306*, 115140.

18 Lancaster, K., Rhodes, T., & Rosengarten, M. (2020). Making evidence and policy in public health emergencies: lessons from COVID‐19 for adaptive evidence‐making and intervention. *Evidence & Policy, 16*(3), 477–490; Lancaster, K., & Rhodes, T. (2022). The thing we call evidence: Towards a situated ontology of evidence in policy In S. Ehlers & S. Esselborn (Eds.), *Evidence in action between science and society: Constructing, validating and contesting knowledge* (pp. 145–164). Routledge.

19 Mannelli, C. (2020). Whose life to save? Scarce resource allocation in the COVID‐19 outbreak. *Journal of Medical Ethics, 46*(6), 364–366.

20 Grasselli, G., Pesenti, A., & Cecconi, M. (2020). Critical care utilisation for the COVID‐19 outbreak in Lombardy, Italy: Early experience and forecast during an emergency response (and associated interview). *JAMA, 323*(16), 1545–1546.

21 Pascoe & Stripling, op. cit. note 16.

22 Elves, C. B., & Herring, J. (2020). Ethical framework for adult social care in COVID‐19. *Journal of Medical Ethics, 46*(10), 662–667.

23 Levy, N., & Savulescu, J. (2021). After the pandemic: New responsibilities. *Public Health Ethics, 14*(2), 120–133.

24 Johnson, J., & Degeling, C. (2019). Does One Health require a novel ethical framework? *Journal of Medical Ethics, 45*(4), 239–243.

25 Lewis & Schuklenk, op. cit. note 11. p. 143.

26 Varia, M., Wilson, S., Sarwal, S., McGeer, A., Gournis, E., & Galanis, E. (2003). Investigation of a nosocomial outbreak of severe acute respiratory syndrome (SARS) in Toronto, Canada. *Canadian Medical Association Journal, 169*(4), 285–292; Cho, S. Y., Kang, J.‐M., Ha, Y. E., Park, G. E., Lee, J. Y., Ko, J.‐H., Lee, J. Y., Kim, J. M., Kang, C. I., Jo, I. J., Ryu, J. G., Choi, J. R., Kim, S., Huh, H. J., Ki, C. S., Kang, E. S., Peck, K. R., Dhong, H. J., Song, J. H., … Kim, Y. J. (2016). MERS‐CoV outbreak following a single patient exposure in an emergency room in South Korea: An epidemiological outbreak study. *The Lancet, 388*(10048), 994–1001; Lyu, W., & Wehby, G. L. (2020). Community use of face masks and COVID‐19: Evidence from a natural experiment of state mandates in the US. *Health Affairs, 39*(8), 1419–1425.

27 Boin & Lodge, op. cit. note 8; Silverstein, A. M. (1981). *Pure politics and impure science: The swine flu affair*. Johns Hopkins University Press.

28 Lancaster, K., et al., op. cit. note 18.

29 Levy & Savulescu, op. cit. note 23; Ansell, C., & Boin, A. (2019). Taming deep uncertainty: The potential of pragmatist principles for understanding and improving strategic crisis management. *Administration & Society, 51*(7), 1079–1112.

30 Degeling, C., Gilbert, G. L., Tambyah, P., Johnson, J., & Lysaght, T. (2020). One health and zoonotic uncertainty in Singapore and Australia: Examining different regimes of precaution in outbreak decision‐making. *Public Health Ethics*, *13*(1), 69–81. https://doi.org/10.1093/phe/phz017

31 Steel, D. (2015). *Philosophy and the precautionary principle*. Cambridge University Press.

32 Douglas, M., Katikireddi, S. V., Taulbut, M., McKee, M., & McCartney, G. (2020). Mitigating the wider health effects of covid‐19 pandemic response. *BMJ, 369*, m1557.

33 Savulescu, J., et al., op. cit. note 8.

34 Elves & Herring, op. cit. note 22.

35 Camporesi, S., & Mori, M. (2021). Ethicists, doctors and triage decisions: Who should decide? And on what basis? *Journal of Medical Ethics, 47*(12), e18.

36 Pascoe & Stripling, op. cit. note 16.

37 Lewis & Schuklenk, op. cit. note 11.

38 National Health Advisory Committee on Immunisation. (2020). *Preliminary guidance on key populations for early COVID‐19 immunisation*. Retrieved May 10, 2023, from http://www.canada.ca/en/public-health/services/immunization/national-advisory-committee-on-immunization-naci/guidance-key-populations-early-covid-19-immunization.html

39 Lewis, J., & Schuklenk, U. (2021). Bioethics met its COVID‐19 Waterloo: The doctor knows best again. *Bioethics, 35*(1), 3–5.

40 Ismail, S. J., Hardy, K., Tunis, M. C., Young, K., Sicard, N., & Quach, C. (2020). A framework for the systematic consideration of ethics, equity, feasibility, and acceptability in vaccine programme recommendations. *Vaccine, 38*(36), 5861–5876.

41 Driedger, S. M., Capurro, G., Jardine, C., & Tustin, J. (2023). Opportunities and perils of public consultation in the creation of COVID‐19 vaccine priority groups. In M. Gattinger (Ed.), *Democratizing risk governance: Bridging science, expertise, deliberation and public values* (pp. 385–413). Springer International Publishing.

42 Herring, J. (2021, February 20). Teachers, and bus drivers ask why they were excluded from the next round of vaccinations. *Calgary Herald*. https://calgaryherald.com/news/local-news/teachers-transit-drivers-among-those-upset-at-exclusion-from-vaccine-list

43 Kholina, K., Harmon, S. H. E., & Graham, J. E. (2022). An equitable vaccine delivery system: Lessons from the COVID‐19 vaccine rollout in Canada. *PLoS ONE, 17*(12), e0279929.

44 Department of Health and Social Care. (2020) *COVID‐19: Ethical framework for adult social care: A framework to support the planning and organisation of adult social care during the coronavirus (COVID‐19) outbreak*. Retrieved May 10, 2023, from https://www.gov.uk/government/publications/covid-19-ethical-framework-for-adult-social-care

45 Elves & Herring, op. cit. note 22.

46 Cordeiro‐Rodrigues, L., & Metz, T. (2021). Afro‐Communitarianism and the role of traditional African healers in the COVID‐19 pandemic. *Public Health Ethics, 14*(1), 59–71; Allinson, R. E. (2020). The primacy of duty and its efficacy in combating COVID‐19. *Public Health Ethics, 13*(2), 179–189.

47 Schmidt, H., Roberts, D. E., & Eneanya, N. D. (2022). Rationing, racism and justice: Advancing the debate around ‘colourblind’ COVID‐19 ventilator allocation. *Journal of Medical Ethics, 48*(2), 126–130.

48 Ibid.; Yu, Q., Salvador, C. E., Melani, I., Berg, M. K., Neblett, E. W., & Kitayama, S. (2021). Racial residential segregation and economic disparity jointly exacerbate COVID‐19 fatality in large American cities. *Annals of the New York Academy of Sciences, 1494*(1), 18–30.

49 Camporesi & Mori, op. cit. note 35.

50 Greenhalgh & Engebretsen, op. cit. note 17.

51 Dewey, J. (1927). *The public and its problems*. H. Holt and Company; Dewey, J. (1995). Does reality possess practical character? In R. B. Goodman (Ed.), *Pragmatism: A contemporary reader* (pp. 79–91). Taylor & Francis Group.

52 Peirce, C. S. (1905). What pragmatism is. *The monist, 15*(2), 161–181.

53 Paavola, S. (2015). Deweyan approaches to abduction. In U. Zackariasson (Ed.), *Action, belief and inquiry—Pragmatist perspectives on science, society and religion* (pp. 230–249). Nordic Pragmatism Network.

54 Fesmire, S. (2003). *John Dewey and moral imagination: Pragmatism in ethics*. Indiana University Press.

55 Dewey (1995), op. cit. note 51.

56 Boin & Lodge, op. cit. note 8.

57 Greenhalgh & Engebretsen, op. cit. note 17, p. 8.

58 Ansell & Boin, op. cit. note 29.

59 Greenhalgh & Engebretsen, op. cit. note 17; Lancaster, K., et al., op. cit. note 18.

60 Sanderson, I. (2009). Intelligent policy‐making for a complex world: Pragmatism, evidence and learning. *Political Studies, 57*(4), 699–719.

61 Samuel, G., & Lucivero, F. (2022). Bringing ethics into governance: The case of the UK COVID‐19 contact tracing app. *International Journal of Health Governance, 27*(2), 180–193.

62 Boin & Lodge, op. cit. note 8; Ansell & Boin, op. cit. note 29.

63 Sanderson, op. cit. note 60.

64 Dahlquist, M., & Kugelberg, H. D. (2021). Public justification and expert disagreement over non‐pharmaceutical interventions for the COVID‐19 pandemic. *Journal of Medical Ethics*, *49*(1), 9–13. https://doi.org/10.1136/medethics-2021-107671

65 Boin, A., & Lodge, M. (2021b). The attractions and limitations of pragmatist crisis management: A discussion in light of COVID‐19 experiences. *LSE Public Policy Review, 1*, 9.

66 Green, J., Fischer, E. F., Fitzgerald, D., Harvey, T. S., & Thomas, F. (2022). The publics of public health: learning from COVID‐19. *Critical Public Health*, *32*(5), 592–599. https://doi.org/10.1080/09581596.2022.2077701; Lancaster, K., et al., op. cit. note 18.

67 Green, J., et al., op. cit. note 66; Pickersgill, M., & Smith, M. (2021). Expertise from the humanities and social sciences is essential for governmental responses to COVID‐19. *Journal of Global Health, 11*, 0308. https://doi.org/10.7189/jogh.11.03081

68 Guttman, N., & Lev, E. (2021). Ethical issues in COVID‐19 communication to mitigate the pandemic: Dilemmas and practical implications. *Health Communication, 36*(1), 116–123.

69 Emanuel, E. J., et al., op. cit. note 16, p. 1544.

70 Dewey (1927) op. cit. note 51.

